# A systematic review and meta-analysis of outcomes following active surveillance, surgery and radiotherapy of meningiomas in NF2-related schwannomatosis

**DOI:** 10.1093/noajnl/vdag022

**Published:** 2026-02-16

**Authors:** Jack Sheppard, Siddarth Kannan, Jane Halliday, Scott Rutherford, Tim Lavin, Claire Forde, Miriam J Smith, Gareth Evans, Andrew T King, Abdurrahman I Islim, Omar N Pathmanaban

**Affiliations:** Manchester Centre for Clinical Neurosciences, Salford Royal Hospital, Manchester, UK; Division of Neuroscience and Experimental Psychology, School of Biological Sciences, Faculty of Biology, Medicine and Health, University of Manchester, Manchester, UK; Geoffrey Jefferson Brain Research Centre, Northern Care Alliance NHS Foundation Trust, Manchester, UK; UCLan School of Medicine & Dentistry, University of Central Lancashire, Preston, UK; Manchester Centre for Clinical Neurosciences, Salford Royal Hospital, Manchester, UK; Geoffrey Jefferson Brain Research Centre, Northern Care Alliance NHS Foundation Trust, Manchester, UK; Manchester Centre for Clinical Neurosciences, Salford Royal Hospital, Manchester, UK; Geoffrey Jefferson Brain Research Centre, Northern Care Alliance NHS Foundation Trust, Manchester, UK; Manchester Centre for Clinical Neurosciences, Salford Royal Hospital, Manchester, UK; Geoffrey Jefferson Brain Research Centre, Northern Care Alliance NHS Foundation Trust, Manchester, UK; Manchester Centre for Genomic Medicine, Saint Mary’s Hospital, Manchester, UK; Geoffrey Jefferson Brain Research Centre, Northern Care Alliance NHS Foundation Trust, Manchester, UK; Manchester Centre for Genomic Medicine, Saint Mary’s Hospital, Manchester, UK; Geoffrey Jefferson Brain Research Centre, Northern Care Alliance NHS Foundation Trust, Manchester, UK; Division of Evolution, Infection and Genomics, School of Biological Sciences, Faculty of Biology, Medicine and Health, University of Manchester, Manchester, UK; Manchester Centre for Genomic Medicine, Saint Mary’s Hospital, Manchester, UK; Geoffrey Jefferson Brain Research Centre, Northern Care Alliance NHS Foundation Trust, Manchester, UK; Division of Evolution, Infection and Genomics, School of Biological Sciences, Faculty of Biology, Medicine and Health, University of Manchester, Manchester, UK; Manchester Centre for Genomic Medicine, Saint Mary’s Hospital, Manchester, UK; Manchester Centre for Clinical Neurosciences, Salford Royal Hospital, Manchester, UK; Division of Neuroscience and Experimental Psychology, School of Biological Sciences, Faculty of Biology, Medicine and Health, University of Manchester, Manchester, UK; Geoffrey Jefferson Brain Research Centre, Northern Care Alliance NHS Foundation Trust, Manchester, UK; Manchester Centre for Clinical Neurosciences, Salford Royal Hospital, Manchester, UK; Geoffrey Jefferson Brain Research Centre, Northern Care Alliance NHS Foundation Trust, Manchester, UK; Division of Immunology, Immunity to Infection and Respiratory Medicine, School of Biological Sciences, Faculty of Biology, Medicine and Health, University of Manchester, Manchester, UK; Manchester Centre for Clinical Neurosciences, Salford Royal Hospital, Manchester, UK; Division of Neuroscience and Experimental Psychology, School of Biological Sciences, Faculty of Biology, Medicine and Health, University of Manchester, Manchester, UK; Geoffrey Jefferson Brain Research Centre, Northern Care Alliance NHS Foundation Trust, Manchester, UK

**Keywords:** active monitoring, meningioma, NF2-related schwannomatosis, stereotactic radiosurgery, surgery

## Abstract

**Background:**

Meningiomas affect up to 80% of patients with *NF2*-related schwannomatosis during their lifetime. They are managed by active monitoring, surgery, and stereotactic radiosurgery (SRS). This paper aims to synthesize the existing data, evaluate outcomes, and inform future trial design.

**Methods:**

Systematic review and meta-analysis conducted using the PRISMA framework. Six databases were searched from inception to September 2025. Patient demographics, intervention data, and outcomes were collected and pooled analyses performed. Studies were appraised using the NIH quality assessment tool.

**Results:**

Fifteen studies with 937 patients and 3637 meningiomas were included. The pooled proportion of female patients was 59.6% (95% confidence interval [CI]: 55.4-63.7). A total of 2082 tumors were monitored (mean follow-up 5.55-9.18 years) with a weighted mean growth rate of 0.508 cm^3^/year (95% CI: 0.0244-0.992) (available in 748 meningiomas). The weighted pooled proportion of monitored patients who developed *de novo* meningiomas across 3 studies was 24.6% (95% CI: 2.73-58.7). Surgical resection was performed in 203 meningiomas, with an under-reported postintervention follow-up and a pooled risk of tumor recurrence of 12.5% (95% CI: 7.98-17.9). Stereotactic radiosurgery was used in 665 meningiomas. The pooled risk of treated tumor progression was 6.29% (95% CI: 4.57-8.25), median follow-up 3.58 to 9.25 years. The pooled local control rates at 3 and 5 years were 97.1% (95% CI: 94.7-98.8) and 91.2% (95% CI: 88.4-93.6), respectively.

**Conclusion:**

*NF2*-associated meningiomas are challenging to manage due to their multiplicity, high growth rate, and World Health Organization grade. Active monitoring, surgery, and SRS are viable treatment options. Here, we evaluate existing outcome data to guide future trial design.

Key Points

- Actively monitored NF2-associated meningiomas had a weighted mean growth rate of 0.508 cm^3^/year.
- The recurrence risk of surgically resected meningiomas was 12.5%.
- Local control rate following SRS treatment at 3 and 5 years were 97.1% and 91.2%, respectively.

Intracranial meningiomas are the second most common tumor found in patients with *NF2*-related Schwannomatosis (*NF2-*SWN) with a prevalence rate between 45% and 58%,^1^ and a lifetime risk of approximately 80% by the age of 70.^2^ Intracranial meningiomas in patients with *NF2*-SWN, referred to throughout as *NF2*-associated meningiomas, have varied growth rates, but are often of a higher histological grade with a higher rate of recurrence.^1^^,^^3–5^ Moreover, the symptoms associated with intracranial meningiomas are thought to be the presenting symptom in 30% of patients with *NF2*-SWN.^6^ These tumors can be difficult to manage due to their multiplicity, with an average of 5 per patient, and the incidence of other intracranial tumors.^5^ This presents a challenge when proactively choosing which tumors to treat, in order to balance the risks and benefits and limit the number of neurosurgical interventions in their lifetime.^7^

Active monitoring is often the initial management strategy in *NF2*-associated meningiomas. There is no consensus on the active monitoring regimen for meningiomas in *NF2*-SWN patients; however, they may require a shorter follow-up interval when compared with asymptomatic incidental meningiomas, and lifelong surveillance.^8^ The choice of active monitoring can, however, convey a significant risk due to the difficulty in predicting their growth rates and the variability in growth rates of tumors in the same patient.^3^^,^^9^ The Asan Intracranial Meningioma Scoring System has shown it may be of value as a prediction tool for meningioma growth in syndromic cases but is yet to undergo adequate validation for clinical use.^7^^,^^10^ The formation of new tumors during active monitoring may also further complicate the decision-making regarding intervention.^5^

Surgical resection is used to treat symptomatic or enlarging meningiomas, with the aim to maximize safe tumor resection whilst preserving quality-of-life (QoL) and neurological function.^11^ Surgery conveys a risk of new transient or permanent neurological deficit, recurrence of the resected tumor and thromboembolic events.^12^ In *NF2*-SWN patients specifically, there is a high prevalence of wound healing complications due to concurrent use of Bevacizumab and corticosteroids in the medical management of the disease.^13^^,^^14^ Due to these complications and the high likelihood of *NF2*-SWN patients requiring multiple operations in their lifetime, the decision to proceed with surgical intervention is complex, with a higher threshold for operating in these patients.^15^

Stereotactic radiosurgery (SRS) may be a favorable intervention in *NF2*-SWN patients, as they often have multiple targetable tumors^4^ and it can be used as an adjuvant therapy to limit local recurrence or progression after surgery.^11^ Radiation-associated side effects, such as radiation necrosis and peri-tumoral edema, have been reported at a similar rate to that observed in sporadic meningiomas.^16^ However, the local control rate of *NF2*-associated meningiomas is lower than sporadic meningiomas due to their aggressiveness and potentially decreased susceptibility to SRS.^17^^,^^18^ Furthermore, there is a risk that the radiation could induce, accelerate, or transform intracranial tumors; especially in *NF2*-SWN patients who have tumor suppressor gene mutations.^19^ Radiation treatment of vestibular schwannoma or a meningioma in 266 patients with *NF2*-SWN conferred an absolute excess malignancy risk of 5% compared with a non-radiated matched control group.^20^ In other studies, malignant transformation of *NF2*-associated meningiomas treated with SRS has not been observed,^21–23^ although the follow-up duration in these studies may not be sufficient to observe these potential radiation-associated complications.

Due to the limitations of existing management strategies for *NF2*-associated meningiomas, there has been a drive toward developing novel therapeutic agents that can reduce intracranial tumor burden and limit progression in a noninvasive manner. The INTUITT-*NF2* trial has showed promising results with the tyrosine-kinase inhibitor Brigatinib conveying broad anti-tumor activity across 40 patients with no significant adverse events.^24^ There are multiple ongoing clinical studies aiming to assess the safety and efficacy of potential therapeutic agents such as the POPLAR-*NF2*^25^ and RETREAT^26^ trials. However, at present there are no clinical benchmarks to compare new treatments against current management strategies. Here, we aim to synthesize and evaluate the existing data on the 3 main management strategies to provide a benchmark for clinical outcomes in ongoing and future clinical trials.

## Methods

### Aims and Objectives

The aim of this study was to synthesize the outcomes of active monitoring, SRS, and surgery in the management of *NF2*-associated meningiomas in order to provide a benchmark for future clinical trials and studies.

### Systematic Search Strategy

This study was conducted according to the Preferred Reporting Items for Systematic Reviews and Meta-Analyses (PRISMA) methodological framework^27^ and the protocol was registered on the PROSPERO research repository (CRD42024566523). Our search examined MEDLINE, EMBASE, Web of Science, Cochrane Library, and Scopus from inception until September 1, 2025. To scrutinize clinical trials, the same search strategy was also applied to the WHO International Clinical Trials Registry Platform and UK Clinical Trials Gateways from inception until September 1, 2025. These searches were restricted to studies published in English. The search comprised MeSH terms and free-text keywords identified from existing literature and guidelines related to *NF2*-related schwannomatosis and meningiomas. The search terms included: “Meningioma*” AND “Neurofibromatosis 2” OR “*NF2*” OR “Schwannomatosis.” For completion, we screened the references of all included articles for any relevant studies.

### Inclusion and Exclusion Criteria

Studies were included if they met following criteria:

- Randomized controlled trials, retrospective or prospective case series, or cohort.
- Conducted in human populations of any gender or nationality.
- Subjects must have *NF2*-related schwannomatosis diagnosed by clinical, radiological, and/or genetic assessment.
- Subjects must have one or more meningiomas diagnosed by radiological and/or histological criteria.
- Reported quantitative data regarding the clinical outcomes of the intervention(s) assessed, including tumor growth rate, *de novo* tumor formation during follow-up, local or distant recurrence following intervention, postintervention complications, quality-of-life outcomes, progression-free survival, and overall mortality.

We excluded studies that

- Included fewer than 10 cases in the documented case series.
- Were individual case reports with/without a review of the literature.
- Included cases that were part of an overlapping cohort with another study.
- Provided insufficient quantitative data regarding the variables under investigation.
- Were published in a language other than English.

### Data Extraction

Following de-duplication of the search results, titles and abstracts were screened separately by 2 independent reviewers (J.S. and S.K.) according to the prescribed inclusion/exclusion criteria. All conflicting decisions regarding inclusion or exclusion were discussed with a third reviewer (A.I.I.) before a final decision was made. A standardized data collection spreadsheet was synthesized to facilitate data extraction. Data were extracted including the following variables: author, year, study design, patient cohort demographics (including number of patients in cohort, average age, gender split) diagnostic criteria for *NF2-*SWN, radiological data regarding the meningioma (such as number, location, and tumor growth rate), and length of follow-up. In studies describing the surgical management of their meningioma, we extracted data regarding the degree of surgical resection, World Health Organization (WHO) grading, postoperative complications and recurrence. Following our abstract screening process, no fractionated or hypofractionated radiotherapy studies met the inclusion criteria, with only studies assessing the outcomes of SRS being included in our analysis. For these studies, we extracted data regarding SRS treatment plan, and progression-free survival. Overall survival and outcomes related to function and quality-of-life were extracted regardless of intervention.

### Quality and Bias Assessment

Each included study was appraised using the NIH Quality Assessment Tool for Observational, Cohort and Cross-Sectional Studies by two authors (S.K. and J.S.). This tool assesses the interval validity of studies by considering the risk of biases (including selection, measurement, and information) as well as confounding.^28^ The final assessment of the strength of evidence for each outcome was calculated utilizing the Grading of Recommendations, Assessment, Development and Evaluations (GRADE) framework. Risk of bias was determined by the NIH Quality Assessment Tool. Heterogeneity was scored according to the *I*^2^ statistic value. Directness was determined by assessing the included studies according to the PICO framework, where studies were penalized for inconsistent outcome definitions. Precision was determined by assessing the 95% confidence interval (CI). Reporting bias was determined by assessment of Harbord and Begg test results,^29^^,^^30^ as well as visual assessment of the funnel plot (range: −1 to 0). The overall score for each outcome was determined as high (≥ 4 points), moderate (3 points), low (2 points), or very low (≤ 1 point).

### Data Synthesis and Analysis

For each study, the number of meningiomas was reported and the location was classified into skull base and non-skull base. The final number of patients was calculated by taking the total number of patients at the start of the follow-up period and subtracting the number of patients who had died during the follow-up period. The final number of meningiomas was calculated with the following equation:


F=(I+Dn)-R

*Equation 1: F = Final Number of Meningiomas, I = Initial Number of Meningiomas, Dn = Number of De Novo Meningiomas, R = Number of Resected Meningiomas*

Meningioma growth rate was recorded as per each study, and the volumes were converted into cm^3^/year format. *De novo* tumors were defined in the active monitoring group as new tumors arising during the follow-up period. The surgically resected meningiomas which were subjected to histological analysis were then classified by their WHO grading. Local recurrence occurred when a tumor remnant progressed after surgical resection. The SRS treatment characteristics, including prescription dose, target volume and fraction, were recorded. In the SRS treatment cohort, *de novo* tumors were defined as new tumors arising outside of the treatment margin. The definition of tumor progression and local control failure varied between studies analyzing patients treated with SRS.

Study-level data were collected and displayed as a number (percent), mean (standard deviation [SD]) or median (range), where appropriate. Meta-analysis of the data was conducted where an outcome variable was uniformly reported across > 2 studies. The pooled proportions (95% CI) of characteristics and outcome measures were calculated using StatsDirect v3.0. Heterogeneity between the analyzed studies was estimated using the *I*^2^ and Cochrane *Q* statistics. An *I*^2^ value of ≤ 25% was deemed low and ≥ 75% as high levels of heterogeneity. When the *I*^2^ value fell in between 25% and 75%, the Cochrane *Q* statistic was used, with a significant value of *P *< 0.05 suggesting high levels of heterogeneity. When heterogeneity was high, a random-effects model was chosen utilizing the DerSimonian-Laird method. A fixed-effect model utilizing the Inverse Variance method was used when heterogeneity was low.

## Results

### Characteristics of Included Studies

**Figure 1.**
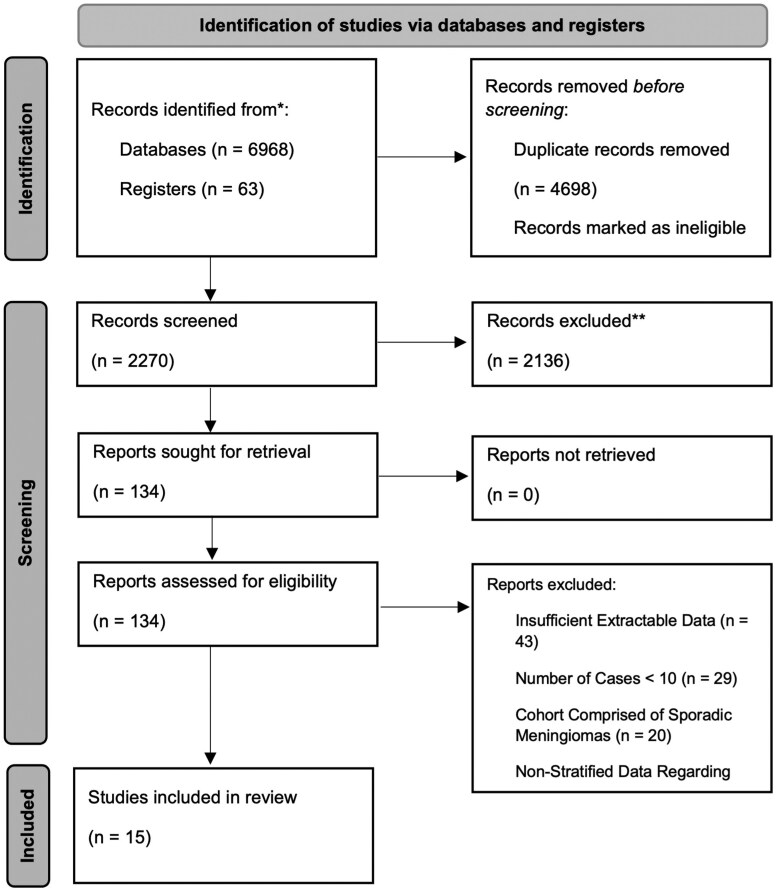
PRISMA flow diagram summarizing the identification, screening, eligibility assessment, and inclusion of studies in the systematic review.^27^

Figure 1 summarizes the study selection protocol. The pooled database search yielded 7031 articles. Following de-duplication, 2270 titles were screened for eligibility by 2 independent reviewers. After resolving any conflicts, 134 studies were selected for full text extraction and review, of which 15 fulfilled the inclusion criteria, and were eligible for data extraction. Table 1 summarizes the key characteristics of the included studies.

**Table 1.** Characteristics of studies included in systematic review

First author, y	No of patients, no of meningiomas	Mean age at diagnosis of NF2-SWN, mean age at treatment (y)	Male/female ratio	*NF2* genetic severity score, no of patients with each mutation	No of patients with single meningioma, no of patients with multiple meningiomas	Meningioma size	Location of meningiomas	Treatment outcomes described (no of meningiomas)	Follow-up duration (y)	Overall survival (%)	Final no of patients	Final no of meningiomas
**Aboukais R, 2013** ^32^	34, 100	26, NR	18, 16	Severe 3-19	15, 19	NR	Skull Base - 6 Non-Skull Base - 19^g^	Active Monitoring - 75 Surgery - 25	9.18 (Mean)	NR	34	77 (25 resected, 2 de novo)
**Birckhead B, 2016** ^22^	15, 113	24, 33.2	6, 9	NR	2, 13	NR	Skull Base - 27 Non-Skull Base—35^g^	SRS - 62	8.58 (Mean Imaging) 9.25 (Mean Clinical)	73.0	11	128 (15 de novo)
**Champeaux-Depond C, 2020** ^36^	184, 315	NR, 40 (Median)	78, 106	NR	120, 64	NR	Skull Base - 68 Non-Skull Base - 116^g^	SRS—25^h^	6.3 (Median)	84.8	156	NR
**Dirks M, 2012** ^3^	17 (13)^a^, 139	33.2^e^, NR	9, 8^e^	NR	NR	NR	Skull Base - 31 Non-Skull Base—108	Active Monitoring - 135 SRS - 2 Surgery - 7	9.5^i^ (Mean)	NR	13	132 (7 resected)
**Evers S, 2015** ^9^	21, 207	28.5^b^, NR	8, 13	NR	NR	Volume - 0.3cm3 (Median)	Skull Base - 31 Non-Skull Base - 176	Active Monitoring - 191 SRS - 5 Surgery - 11	5.55 (Mean)	71.4	15	199 (11 resected)
**Gao F, 2019** ^23^	35, 99	NR, 40 (Median)	10, 25	NR	12, 23	NR	Skull Base - 25 Non-Skull Base - 74	SRS - 99	8 (Median)	82.9	35	95 (4 resected after local control failure)
**Goutagny S, 2012** ^5^	74, 287	29.1, NR	26, 48	Tissue Mosaic 1A - 6 Tissue Mosaic 1B - 1 Classic 2A- 17 Severe 3 - 18	NR	Max diameter - 1.43cm (Mean) Volume - 2.4cm3 (Mean, N = 68)	Skull Base - 71 Non-Skull Base - 216	Surgery - 56	9.18^j^ (Mean)	88.0	65	262 (56 resected, 25 de novo, 6 local recurrence)
**Jaoude S, 2021** ^7^	92, 358	27.5^c^, NR	37, 55	Tissue Mosaic 1 - 20 Classic 2 - 3 Severe 3-11^f^	16, 76	Volume - 5.43cm3 (Mean)	Skull Base - 103 Non-Skull Base - 255	Surgery - 66 SRS - 2 SRS + Surgery - 5	15.5^j^ (Mean)	NR	92	426 (66 resected, 138 de novo, 6 local recurrence)
**Li P, 2020** ^33^	452 (289)^a^, 1020	NR	NR	NR	NR	Volume - 0.969cm3 (Median, N = 148)	Skull Base - 154 Non-Skull Base - 866	Active Monitoring - 148	NR	NR	289	1059 (39 de novo)
**Liu A, 2015** ^21^	12, 125	NR, 31 (Median)	2, 10	NR	0, 12	Max Long-Axis - 1.6cm (Median, N = 125)	Skull Base - 31 Non-Skull Base - 94	SRS - 87	3.58^k^ (Median)	66.6	8	122 (3 resected after local control failure)
**Mohammed N, 2022** ^37^	39, 386	30 (Median), 38 (Median)	NR	NR	NR	NR	Skull Base - 52 Non-Skull Base - 150	SRS - 204	8.5^k^ (Median)	NR	39	384 (2 resected after tumor progression)
**Nowak A, 2015** ^34^	21, 118	20.8^d^, NR	9, 12	NR	5, 16	Volume - 3.4cm3 (Mean)	Skull Base - 32 Non-Skull Base - 86	Surgery - 21	6.5 (Mean Imaging)	NR	21	112 (21 resected, 12 de novo, 3 local recurrence)
**Oyem P, 2022** ^8^	48, 137	39.5^b^, NR	NR	NR	NR	Volume - 5.2cm3 (Mean)	Skull Base - 22 Non-Skull Base - 114	Active Monitoring - 106 Surgery - 20 Radiotherapy - 11	2.67 (Median Imaging)	NR	48	117 (20 resected)
**Ruggieri M, 2005** ^35^	24 (16)^a^, 20	7.79, NR	7, 9	N/A DNA analysis performed in 9 patients: No mutation found - 4 Substitution—4 (Splicing—2, Not Sequenced - 2) Deletion resulting in frameshift - 1	12, 4	NR	Skull Base - 8 Non-Skull Base - 12	Surgery - 3	NR	93.8	15	16 (4 resected)
**Wei Z, 2024** ^38^	45, 213	42.4, 51.6	20, 25	NR	NR	NR	Skull Base - 49 Non-Skull Base - 164	SRS - 213	4.36 ± 3.82 (Mean) 5.29 (Median) Range 0.5-22.3	60%	27	NR

a Number of patients with intracranial meningioma.

b Mean at Initial Scan.

c Mean at Meningioma Diagnosis.

d Mean at First Symptoms.

e Total Population Including Patients without Meningiomas.

f Genetic analysis performed in 34 patients with at least 1 rapidly growing meningioma.

g Location of Treated Meningiomas Only.

h Number refers to patients as opposed to meningiomas.

i Mean follow-up of all tumors including meningiomas.

j Mean follow-up after diagnosis.

k Median follow-up following stereotactic radiosurgery.

### Baseline Patient Characteristics

Fifteen studies with 937 patients were included. The mean age at *NF2*-SWN diagnosis was not consistently reported in the included studies. The range of mean age at diagnosis across 5 studies was 7.79 to 42.4 years. The pooled weighted proportion of patients who were female was 59.6% (95% CI: 55.5-63.7, *I*^2^ = 0.00). Included were 937 patients with a total of 3637 meningiomas. Most patients harbored multiple meningiomas (67.0%, 95% CI: 47.0-84.2, *I*^2^ = 0.930). The average number of tumors per patient was not routinely reported. The pooled weighted proportion of meningiomas which were located at the skull base was 22.9% (95% CI: 19.2-26.8, *I*^2^ = 0.806). Three studies reported the results of a genetic analysis in 70 patients with sequencing data available in 65 patients. The *NF2* genetic severity score was reported in 3 studies with 48 patients classified as ‘Severe 3’, which corresponds with full *NF2* truncating mutation at exons 2 to 13.^31^

### Active Monitoring

A total of 2082 meningiomas were actively monitored, with the summary of tumor characteristics displayed in Table 2.^3^^,^^7–9^^,^^32–34^ The mean follow-up period ranged from 5.55 to 9.18 years across 3 studies. The remaining 4 studies reported follow-up duration as a median, mean after *NF2*-SWN diagnosis or as a mean including patients without meningiomas. The follow-up imaging protocol, where reported, comprised annual brain and spine magnetic resonance imaging (MRI). The weighted mean growth rate in 748 meningiomas, subject to analysis, was 0.508 cm^3^/year (95% CI: 0.0244-0.992). Two studies reported the proportion of patients developing symptoms secondary to meningioma growth, and this ranged from 6.78 to 10%. The pooled weighted proportion of meningiomas that progressed to require treatment was 14.1% (95% CI: 8.70-20.4, *I*^2^ = 0.92) across 7 studies. There were 259 *de novo* meningiomas reported in 5 studies during the follow-up period; the weighted pooled proportion of patients who developed *de novo* meningiomas across 3 studies was 24.6% (95% CI: 2.73-58.7, *I*^2^ = 0.950).

**Table 2.** Summary of the characteristics of actively monitored meningiomas

First author, y	No of meningiomas	Initial volume (cm^3^)	Final volume (cm^3^)	Growth rate (cm^3^/y)	De novo meningiomas, no of patients	% of patients that develop symptoms	% of meningiomas that progress to require treatment	Follow-up duration (y)
**Aboukais R, 2013** ^32^	100	NR	NR	NR	4, NR	10	25	9.18 (Mean)
**Dirks M, 2012** ^3^	71 (Initial)^a^ 139 (Total)	NR	NR	0.4 ± 0.8 (Mean) (N = 135)	66, 11	NR	5	9.5 ± 4.8^c^ (Mean)
**Evers S, 2015** ^9^	207 (Analyzed)^b^ 210 (Total)	0.30 (Mean)	0.88 cm3 (Mean)	0.09 (Median) (N = 207)	NR, NR	NR	7.62	5.55 ± 2.48 (Mean)
**Jaoude S, 2021** ^7^	358	5.43 (Mean)	NR	2.24 (Mean) (N = 358)	138, NR	NR	18	15.5 (Mean)^d^
**Li P, 2020** ^33^	225 (Analyzed)^b^ 1020 (Total)	0.97 (Median)	NR	0.218 (Median) (N = 148)	39, 24	NR	7.55	NR
**Nowak A, 2015** ^34^	118	3.4 (Mean)	NR	0.5 (Mean) (N = 118)	12, 6	6.78	18	6.5 ± 3.7 (Mean Imaging)
**Oyem P, 2022** ^8^	137	5.23 (Mean)	NR	0.73 (Mean) (N = 137)	NR	NR	22.6	2.67 (Median)

a Number of tumors at the start of analysis, with total including De Novo meningiomas.

b Number of tumors subjected to growth analysis.

c Mean follow-up for all tumors including meningiomas.

d Mean follow-up after diagnosis.

### Surgical Management

A total of 203 meningiomas were surgically resected, with the summary of surgical treated patients shown in Table 3.^5^^,^^7–9^^,^^32^^,^^34^^,^^35^ Of the resected tumors with a histological diagnosis (N = 159), the pooled weighted proportion of “high-grade” meningiomas (WHO Grade 2 and 3) was 26.5% (95% CI: 12.5-43.5, *I*^2^ = 0.810). The extent of tumor resection was not consistently reported across the included studies. Tumor recurrence was reported in 4 studies, with a pooled weighted proportion of 12.5% (95% CI: 7.98-17.9, *I*^2^ = 0.00). *De novo* meningioma development during follow-up was reported in 4 studies, with a total of 175 *de novo* tumors. Three studies reported postoperative complications, with a total of 9 patients experiencing a neurological deficit, 5 of which were reported as permanent deficits. The weighted pooled proportion of patients that experienced postoperative complications was 15.0% (95% CI: 0.09-41.7, *I*^2^ = 0.820). Postoperative follow-up duration was only reported in one study by Nowak et al, with a mean follow-up of 3.10 years.^34^ Otherwise, follow-up was reported as a total duration following diagnosis of *NF2*-SWN.

**Table 3.** Summary of the characteristics of surgically resected meningiomas

First author, y	Total no of meningiomas, no of surgically resected meningiomas	WHO grade			Local recurrence risk (%)	De novo meningioma development, no of patients with de novo meningiomas	Complication rate (%), type of complication	Follow-up duration after intervention (y)
		I	II	III				
**Aboukais R, 2013** ^32^	100, 25	20	5	0	20	0, NR	0	NR^a^
**Evers S, 2015** ^9^	210, 11	NR	NR	NR	NR	NR, NR	NR	NR^a^
**Goutagny S, 2012** ^5^	287, 56	29^b^	10^b^	2^b^	10.7	25, 12	7 Worsening of neurological status - 4	NR^a^
**Jaoude S, 2021** ^7^	358, 66	25^c^	26^c^	1^c^	9.09	138, NR	NR	NR^a^
**Nowak A, 2015** ^34^	118, 21	20	1	0	14.3	12, 6	45.4 Cranial nerve IX and X deficits - 3 Hemiparesis - 1 Unilateral blindness - 1	3.1 (Mean)
**Oyem P, 2022** ^8^	137, 20	15	4	1	NR	NR	NR	NR^a^
**Ruggieri M, 2005** ^35^	20, 4	NR	NR	NR	NR	NR	NR	NR^a^

a Follow-up duration after intervention not documented, overall follow-up provided in Table 1.

b Histological analysis performed in a total of 41 surgically resected meningiomas.

c Histological analysis performed in a total of 52 surgically resected meningiomas.

### Radiotherapy and Radiosurgery

A total of 665 meningiomas were treated using SRS; the detailed description of tumors treated with SRS is shown in Table 4.^8^^,^^9^^,^^21–23^,^36–38^ The median prescription dose at tumor margin ranged from 12 to 16 Gy and the median maximum tumor dose ranged from 24 to 32 Gy. The median tumor volume treated ranged from 1.33 to 6.80 cm^3^ with a range of 0.100 to 68.4cm^3^ across 3 studies. The pooled weighted proportion of patients who experienced post-treatment complications was 14.6% (95% CI: 9.39-20.7, *I*^2^ = 0.728); peritumoral edema was the most common complication followed by radiation necrosis, with an incidence of 9.59% (14/146) and 5.48% (8/146) respectively. *De novo* meningioma development was reported in 3 studies, for a total of 69 tumors in 38 patients. Across 5 studies, progression was reported in 41/665 of treated meningiomas; the pooled weighted proportion of progression in tumors treated with SRS was 6.29% (95% CI: 4.57-8.25, *I*^2^ = 0.353). The pooled weighted proportion of the local control rate at 3 and 5 years was 97.1% (95% CI: 94.7-98.8, *I*^2^ = 0.00) and 91.2% (95% CI: 88.4-93.6, I^2^ = 0.00), respectively. There was no malignant transformation reported in the treated tumors across 5 studies. The postintervention follow-up was reported in 5 studies; a weighted mean could not be calculated as only one study reported a mean follow-up duration. The range of median postintervention follow-up was 3.58 to 9.25 years.

**Table 4.** Summary of the characteristics of meningiomas treated with stereotactic radiosurgery

First author, ye	Total no of meningiomas, no of meningiomas treated with radiosurgery	Prescription dose at tumor margin (Gy)	Maximum tumor dose (Gy)	No of fractions (mean)	Tumor volume treated (cc)	De novo meningioma development, no of patients with de novo meningiomas	percentage of patients experiencing complications (%), type of complication and incidence	Progression risk (%)	1-year local control (%)	3-year local control (%)	5-year local control (%)	10-year local control (%)	No of malignant transformations	Follow-up after intervention (y)
**Birckhead B, 2016** ^22^	113, 62	16 (Median) Range: 13-20	32 (Median) Range: 24-40	1	6.8 (Median) Range: 0.6-68.4	15, 4	26.7 Peritumoral edema - 2 Radiation necrosis - 1 Cavernous malformation - 1	3.23	NR	98	96	96	0	8.58 (Mean Imaging) 9.25 (Mean Clinical)
**Champeaux-Depond C, 2020** ^36^	315, NR	NR	NR	NR	NR	NR, NR	NR	NR	NR	NR	NR	NR	NR	NR
**Ever S, 2015** ^9^	210, 5	NR	NR	NR	NR	NR, NR	NR	NR	NR	NR	NR	NR	NR	NR
**Gao F, 2019** ^23^	99, 99	13 (Median) Range: 12-15	26 (Median) Range: 24-30	1	6.8 (Median) Range: 0.6-40	38, 18	17.1 Radiation necrosis - 6 Peritumoral edema - 6	4.04	100	97.1	90.6	NR	0	8 (Median)
**Liu A, 2015** ^21^	125, 87	12 (Median) Range: 10-15	NR	NR	NR	16, 6	50 Peritumoral edema - 1	4.60	100	98	92	NR	0	3.58 (Median)
**Mohammed N, 2022** ^37^	386, 204	12.5 (Median) Range: 10-25	26 (Median) Range: 20-50	1	1.33 (Median) Range: 0.1-21.2	NR, NR	10.3 Peritumoral edema - 3 Radiation necrosis - 1	4.9	NR	NR	NR	NR	0	8.5 (Median)
**Oyem P, 2022** ^8^	137, 11	NR	NR	NR	NR	NR, NR	NR	NR	NR	NR	NR	NR	NR	NR
**Wei Z, 2024** ^38^	213, 213	13 (Median) Range: 9-16	24 (Median) Range: 7.69-34.2	NR	NR	NR, 20	4.44 Peritumoral edema - 2	9.86	NR	NR	90.2	84.5	0	5.29 (Median)

### Functional Outcomes

From our 15 included studies, only 3 reported measures of functional status. Wei et al reported a median post-SRS Karnofsky Performance Status (KPS) Score of 90 (range = 5-100), with 19 (42.2%) showing improvement, 24 (53.3%) remaining static, and 2 (4.44%) worsening compared with pre-treatment.^38^ Dirks et al reported a change in the median KPS over the follow-up period from 90 to 80, with the range of scores remaining at 60 to 90. However, this measurement was for the entire cohort of 17 patients, including the 4 patients without intracranial meningiomas.^3^ Elsewhere, Gao et al reported a median KPS of 80 (range: 60-90) in their cohort at last follow-up.^23^

### Quality and Bias Assessment

The breakdown of the quality assessment conducted on the 15 included studies is summarized in Table S1. Twelve studies were rated “good,” three studies were rated “fair,” and no studies were rated “poor.” The results of the GRADE framework analysis are shown in Table 5. The quality of evidence informing the pooled risk of each outcome was “very low” in 6 outcomes, “low” in 2 outcomes and “moderate” in 1 outcome. Directness was penalized in the local recurrence after surgery and tumor progression after SRS as there was no unifying definition of local recurrence or tumor progression among the included studies. Reporting bias could not be calculated in 3 outcomes because there were 3 or less included studies.

**Table 5.** Level of evidence informing the outcomes for Active monitoring, Surgery and Stereotactic Radiosurgery, assessing utilizing the GRADE framework

s	Outcome	Pooled risk (%) (95% CI)	No of studies	No of patients/meningiomas	Quality assessment						
					Type of evidence	Risk of bias	Heterogeneity	Directness	Precision	Reporting bias	Overall
**Active Monitoring**	Tumor Progression to Require Treatment	14.1^a^ (8.70-20.4)	7	2082^e^	+2	−1	−1	0	0	0	Very Low ⨁◯◯◯
	De Novo Tumor Formation	24.6^b^ (2.73-58.7)	3	503^d^	+2	−1	−1	0	−1	NA^h^	Very Low ⨁◯◯◯
	Annual Growth Rate	0.508^c^ (0.0244-0.992)	4	748^d^	+2	−1	−1	0	−1	NA^i^	Very Low ⨁◯◯◯
**Surgery**	Local Tumor Recurrence	12.5^a^ (7.98-17.9)	4	203^e^	+2	0	+1	−1^f^	0	0	Low ⨁⨁◯◯
	Post-operative Complications	15^b^ (0.09-41.7)	3	62^d^	+2	0	−1	0	−1	NA^h^	Very Low ⨁◯◯◯
**Stereotactic Radiosurgery**	Post-SRS Complications	14.6^b^ (9.39-20.7)	5	146^d^	+2	−1	0	0	0	−1	Very Low ⨁◯◯◯
	Tumor Progression	6.2^a^ (4.70-8.20)	5	665^e^	+2	−1	0	−1^g^	0	0	Very Low ⨁◯◯◯
	Local Control 3-Years	97.1^b^ (94.7-98.8)	3	62^d^	+2	0	+1	0	0	NA^h^	Moderate ⨁⨁⨁◯
	Local Control 5-Years	91.2^b^ (88.4-93.6)	4	107^d^	+2	−1	+1	0	0	0	Low ⨁⨁◯◯

NIH: National Institute of Health. PICO: Population, Intervention, Comparison, Outcome. CI: Confidence Interval. SRS: Stereotactic Radiosurgery. NA: Not applicable.

Type of evidence was based on the predominant design of the included studies; +2 refers to observational, cohort and cross-sectional studies, +4 refers to randomized controlled trials. Risk of bias reflects the quality of the methodological and statistical methods in each study that outcome data was derived from, as determined by the NIH Quality Assessment Tool (range: − 2 to 0). Heterogeneity was scored according to the I_2_ statistic value; low (≤ 25%) = + 1, moderate (25%≤ *n *≤ 75%) = 0 and high (≥ 75%) = − 1. Directness was determined by assessing the included studies according to the PICO framework, where studies were penalized for inconsistent outcome definitions (range: −2 to 0). Precision was determined by assessing the 95% CI (range: − 1 to 0). Reporting bias was determined by assessment of Harbord and Begg test results, as well as visual assessment of the funnel plot (range: −1 to 0). The overall score for each outcome was determined as high (≥ 4 points), moderate (3 points), low (2 points) or very low (≤ 1 point). The score is represented visually with each “⊕” symbol corresponding to 1 point, and a caption stating the overall quality of evidence (ie, high, moderate, low, or very low).

a Values are represented as pooled risk per treated tumor.

b Values are represented as pooled risk per patient receiving treatment.

c Value represents weighted mean annual growth rate with 95% CI.

d Number of patients.

e Number of meningiomas.

f No definitive definition of tumor recurrence across the included studies, and the extent of tumor resection was not routinely reported across the studies that used this outcome measure.

g No definitive definition of tumor progression across the included studies.

h Not assessed due to low number of studies.

i Not assessed due to lack of reporting bias data.

## Discussion

Here, we present a comprehensive and up-to-date summary of the existing data on the management strategies for meningiomas in *NF2*-related Schwannomatosis. The field of *NF2*-SWN is rapidly developing, with multiple active clinical trials that are assessing the safety and efficacy of drug therapies including the histone deacetylase (HDAC) inhibitor REC-2282,^25^ targeted cancer therapies Brigatinib and Neratinib^24^ and anti-retroviral treatments Lopinavir and Ritonavir.^26^ Despite this ongoing research, there has been no universal consensus on outcomes that should be used to compare the efficacy of new treatments against existing management strategies. Our initial aim was to provide benchmark outcomes for the 3 major management strategies for *NF2*-associated meningiomas; however, our data analysis and quality appraisal has revealed that the evidence for most outcomes assessed is low or very low certainty. We posit that this relatively low quality of evidence is due to the heterogeneity, risk of methodological bias, and the small population size of included studies. Although we did not meet the original aim of our review, this review still provides a comprehensive analysis and evaluation of the existing outcome data for active surveillance, surgery, and radiotherapy in *NF2*-associated meningiomas.

This review provides the first analysis of all active monitoring data for *NF2*-associated meningiomas, with a total of 2082 tumors monitored over a mean follow-up of 5.55 to 9.18 years. We report an average absolute growth rate of 0.508cm^3^/year in 748 meningiomas, exceeding the growth rates reported in 2 studies of sporadic meningiomas: 0.240 cm³/year (10 patients, 47 months average follow-up) and 0.05 cm³/year (240 tumors, 67 months median follow-up), respectively.^39^^,^^40^ This mean value may be used as a baseline growth rate for comparison with pharmacological interventions in single-arm clinical trials or studies where randomization is not possible. Only 2 studies reported the incidence of meningiomas that developed symptoms during follow-up, ranging between 6.78 and 10%. This is comparable to a series of 608 patients with sporadic meningiomas, which showed 8.1% of patients developed symptoms across a follow-up of 49.5 months.^41^ Despite having a higher absolute growth rate and a similar rate of symptom development, *NF2*-associated meningiomas had a lower progression rate requiring treatment (14.1%) compared to incidental meningiomas (24.8%).^41^ This may be due to the higher threshold for intervention in *NF2-*SWN patients as there are an increased incidence of multiple intracranial tumors and a higher likelihood of requiring multiple interventions during their lifetime.^4^^,^^19^ As reported in Table 5, the outcomes for actively monitored meningiomas showed a high degree of heterogeneity, which may be due to differing radiological follow-up regimens, genetic severity scores or outcome definitions within the included studies. Therefore, these should be interpreted cautiously with this context in mind.

In surgically managed meningiomas, the long-term prognosis is dependent on the extent of surgical resection and imaging findings, such as peri-tumoral edema, which have been shown to be more reliable predictors of tumor recurrence or progression than WHO grade alone.^42^^,^^43^ The extent of surgical resection was not consistently reported, with only Aboukais et al describing the specific Simpson grade for the entire cohort.^32^ Elsewhere, the incidence of gross total surgical resection in *NF2*-associated meningiomas has been reported as 73.7% in 19 cases.^18^ Similarly, a 78.9% gross total resection rate was reported in a single-center study of 1469 meningioma patients.^44^ Our findings show that 26.5% of resected *NF2*-associated meningiomas were “high-grade” (WHO grade 2 and 3), significantly higher than the 4.11% observed in sporadic meningiomas.^44^ The complication rate was reported in 3 studies, with a weighted pooled incidence of 8.82% in 102 patients; however, there was high heterogeneity in this value, which limits its interpretation. This is comparable to the complication rate of 11.8% reported in 533 of surgically resected sporadic meningiomas. We report a pooled weighted local recurrence rate of 12.5% in 168 surgically resected tumors across 4 studies. However, due to the inconsistent reporting of the extent of surgical resection, a known predictor of tumor recurrence, this pooled weighted recurrence rate should be interpreted with caution. The postintervention follow-up was not routinely reported, with most studies reporting overall follow-up with the surgical intervention occurring during this period.

We analyzed 665 *NF2*-associated meningiomas treated with SRS across 8 studies; in 146 patients where data were available, the weighted pooled complication rate was 14.6%. Elsewhere in the literature, the incidence of radiation-toxicity associated side effects has been estimated at between 7% and 17.4%.^45–47^ We report local control rates across three studies of 97.1% and 91.2% at 3 and 5 years, respectively. Our analysis did not include progression-free survival due to inconsistent reporting between studies; however, Habibi et al reported a progression-free survival rate of 96% at 12 months, 95% at 3 years, and 93% at 5 years.^48^ In a meta-analysis of 4229 patients with WHO grade 1 and 2 intracranial meningiomas, the progression-free survival ranged between 91.3% and 100% at 3 years and 78% and 98.9% at 5 years after SRS treatment.^45^ Although *de novo* tumor formation is a potential risk of SRS, a study of 1837 patients treated for arteriovenous malformation (AVM), or benign tumors identified no radiation-induced tumors over 11 264 patient-years of follow-up.^49^ Notably, these patients did not have a genetic predisposition for tumor development. In other papers, the risk of secondary intracranial neoplasm formation after SRS has been reported at between 0.04% and 2.60% when treated similar pathologies.^50^^,^^51^ In this meta-analysis, we were not able to compare the risk of new tumor formation or malignant transformation across management groups. Evans et al showed that radiotherapy conveyed an increase in 20-year and lifetime risk of malignancy/malignant progression in *NF2*-SWN patients.^20^ Pollock et al reported the 15-year malignant transformation risk of 2.40%, with meningioma significantly more likely to transform.^49^

In our analysis, QoL and functional outcomes were not consistently reported, with only 3 studies reporting KPS as a measure of overall measure of function, and only Wei et al and Dirks et al reporting change in KPS before and after intervention.^3^^,^^23^ The limited assessment of QoL and functional outcomes in *NF2*-associated meningiomas is mirrored in sporadic cases. Two studies have demonstrated conflicting results when comparing neurocognitive measures between incidental meningiomas and matched controls^52^^,^^53^; and no studies have evaluated the impact of interventions on QoL outcomes.^41^

This meta-analysis has several limitations. The studies included in our analysis were retrospective and published over a period where multiple advancements in the management of *NF2*-associated meningiomas had been made.

During our statistical analysis, we found that several outcomes (such as tumor progression during surveillance, *de novo* tumor formation and proportion of WHO 2/3 meningiomas) showed high heterogeneity between studies. This may reflect variability in the specific *NF2* mutations, center-specific *NF2*-SWN management strategies, and prior interventions received by the patients. To counteract this heterogeneity, we considered performing subgroup analyses; however, this was not possible as the data were not stratified to pre-intervention characteristics. We also observed heterogeneity in the reporting of tumor location, growth rate, and postintervention complications, as well as the definition of tumor progression and local control failure in studies analyzing patients treated with SRS. This highlights the need for a consensus definition of these important outcomes. The lack of data regarding postintervention follow-up limits the interpretation and comparison of our data, especially regarding tumor progression and/or recurrence. This highlights the requirement for further longitudinal studies that assess *NF2*-associated meningioma recurrence rate over time following surgical resection. Furthermore, we identified several areas where data are lacking in the existing literature, including the genetic analysis of included meningiomas as well as the functional and QoL outcomes that we aimed to interrogate in this review.

## Conclusion

*NF2*-associated meningiomas are challenging to manage due to their comparatively high growth rate, WHO grade, and their multiplicity in patients with *NF2*-related Schwannomatosis. Therefore, regardless of the choice of management strategy, there is a necessity for close monitoring and follow-up to identify local and distant treatment failure as well as postintervention complications. Our review provides the most up-to-date and comprehensive synthesis of existing data on postintervention outcomes in *NF2*-associated meningiomas. Furthermore, we identified important areas of data, such as genetic analysis and functional outcomes, which are lacking in the current literature and require further interrogation in future studies. These findings can be used in the design of future clinical trials, to compare new therapeutic agents or treatments against existing management strategies.

## Supplementary Material

vdag022_Supplementary_Data

## Data Availability

The extracted data and details of the statistical methods used in this study are available from the corresponding author (J.S.) upon reasonable request.

## References

[1] Teranishi Y , Miyawaki S, Nakatochi M, et al Meningiomas in patients with neurofibromatosis type 2 predominantly comprise ‘immunogenic subtype’ tumours characterised by macrophage infiltration. Acta Neuropathol Commun. 2023;11:156. 10.1186/s40478-023-01645-3 37752594 PMC10521403

[2] Smith MJ , Higgs JE, Bowers NL, et al Cranial meningiomas in 411 neurofibromatosis type 2 (NF2) patients with proven gene mutations: clear positional effect of mutations, but absence of female severity effect on age at onset. J Med Genet. 2011;48:261-265. 10.1136/jmg.2010.085241 21278391

[3] Dirks MS , Butman JA, Kim HJ, et al Long-term natural history of neurofibromatosis type 2–associated intracranial tumors. J Neurosurg. 2012;117:109-117. 10.3171/2012.3.JNS111649 22503123 PMC4749021

[4] Bachir S , Shah S, Shapiro S, et al Neurofibromatosis type 2 (NF2) and the implications for vestibular schwannoma and meningioma pathogenesis. Int J Mol Sci. 2021;22:1-12. 10.3390/ijms22020690 PMC7828193 33445724

[5] Goutagny S , Bah AB, Henin D, et al Long-term follow-up of 287 meningiomas in neurofibromatosis type 2 patients: clinical, radiological, and molecular features. Neuro Oncol. 2012;14:1090-1096. 10.1093/neuonc/nos129 22711605 PMC3408259

[6] Evans DG , Huson SM, Donnai D, et al A clinical study of type 2 neurofibromatosis. Q J Med. 1992;84:603-618. http://www.ncbi.nlm.nih.gov/pubmed/1484939[PMC][1484939] 1484939

[7] Jaoude SA , Peyre M, Degos V, et al Validation of a scoring system to evaluate the risk of rapid growth of intracranial meningiomas in neurofibromatosis type 2 patients. J Neurosurg. 2021;134 5 1377-1385. 10.3171/2020.3.JNS192382 32442973

[8] Oyem PC , de Andrade EJ, Soni P, et al Natural history and volumetric analysis of meningiomas in neurofibromatosis type 2. Neurosurg Focus. 2022;52:E5-6. 10.3171/2022.2.FOCUS21779 35535826

[9] Evers S , Verbaan D, Sanchez E, Peerdeman S. 3D volumetric measurement of neurofibromatosis type 2-associated meningiomas: association between tumor location and growth rate. World Neurosurg. 2015;84:1062-1069. 10.1016/j.wneu.2015.05.068 26087434

[10] Lee EJ , Kim JH, Park ES, et al A novel weighted scoring system for estimating the risk of rapid growth in untreated intracranial meningiomas. J Neurosurg. 2017;127:971-980. 10.3171/2016.9.JNS161669 28084908

[11] Goldbrunner R , Stavrinou P, Jenkinson MD, et al EANO guideline on the diagnosis and management of meningiomas. Neuro Oncol. 2021;23:1821-1834. 10.1093/neuonc/noab150 34181733 PMC8563316

[12] Wong JM , Panchmatia JR, Ziewacz JE, et al Patterns in neurosurgical adverse events: intracranial neoplasm surgery. Neurosurg Focus. 2012;33:E16-3. 10.3171/2012.7.FOCUS12183 23116096

[13] Morris KA , Golding JF, Axon PR, et al Bevacizumab in neurofibromatosis type 2 (NF2) related vestibular schwannomas: a nationally coordinated approach to delivery and prospective evaluation. Neurooncol Pract. 2016;3:281-289. 10.1093/nop/npv065 29692918 PMC5909937

[14] Ladha H , Pawar T, Gilbert MR, et al Wound healing complications in brain tumor patients on bevacizumab. J Neurooncol. 2015;124:501-506. 10.1007/s11060-015-1868-0 26298437

[15] Gregory GE , Islim AI, Hannan CJ, et al The clinical, genetic, and immune landscape of meningioma in patients with NF2-schwannomatosis. Neurooncol Adv. 2023;5:i94-i104. 10.1093/noajnl/vdac127 37287576 PMC10243851

[16] Gomes FC , Larcipretti ALL, Mariano, et al GammaKnife radiosurgery for meningiomas in neurofibromatosis type II patients: a systematic review and meta-analysis. Neurosurg Rev. 2025;48:368-368. 10.1007/s10143-025-03519-9 40237947

[17] Santacroce A , Walier M, Régis J, et al Long-term tumor control of benign intracranial meningiomas after radiosurgery in a series of 4565 patients. Neurosurgery. 2012;70:32-39; discussion 39. 10.1227/NEU.0b013e31822d408a 21765282

[18] Nguyen T , Chung LK, Sheppard JP, et al Surgery versus stereotactic radiosurgery for the treatment of multiple meningiomas in neurofibromatosis type 2: illustrative case and systematic review. Neurosurg Rev. 2019;42:85-96. 10.1007/s10143-017-0904-2 28900754

[19] Gareth Evans D. NF2-related schwannomatosis. Gene Reviews. 2023;64:1-13.

[20] Evans DG , Halliday D, Obholzer R, et al Radiation treatment of benign tumors in NF2-related-schwannomatosis: a national study of 266 irradiated patients showing a significant increase in malignancy/malignant progression. Neurooncol Adv. 2023;5:vdad025- 10.1093/noajnl/vdad025 37051330 PMC10084499

[21] Liu A , Kuhn EN, Lucas JT, et al Gamma knife radiosurgery for meningiomas in patients with neurofibromatosis type 2. J Neurosurg. 2015;122:536-542. 10.3171/2014.10.JNS132593 25555193 PMC9168962

[22] Birckhead B , Sio TT, Pollock BE, et al Gamma knife radiosurgery for neurofibromatosis type 2-associated meningiomas: a 22-year patient series. J Neurooncol. 2016;130:553-560. 10.1007/s11060-016-2257-z 27816997

[23] Gao F , Li M, Wang Z, et al Efficacy and safety of gamma knife radiosurgery for meningiomas in patients with neurofibromatosis type 2: a long-term follow-up single-center study. World Neurosurg. 2019;125:e929-e936. 10.1016/j.wneu.2019.01.211 30763746

[24] Plotkin SR. Innovative trial for understanding the impact of targeted therapies in NF2-related schwannomatosis (INTUITT-NF2). Preprint posted online April 30, 2020. https://clinicaltrials.gov/study/NCT04374305

[25] Pharmaceuticals R. Efficacy and safety of REC-2282 in patients with progressive neurofibromatosis type 2 (NF2) mutated meningiomas (POPLAR-NF2). Preprint posted online November 2021. https://clinicaltrials.gov/study/NCT05130866

[26] Campbell S , Hanemann C. A trial to test the use of HIV drugs to treat neurofibromatosis type 2 (NF2) related tumours. http://isrctn.com/. Preprint posted online 2024. 10.1186/ISRCTN10422213

[27] Page MJ , McKenzie JE, Bossuyt PM, et al The PRISMA 2020 statement: an updated guideline for reporting systematic reviews. BMJ. 2021;372:n71. 10.1136/bmj.n71 33782057 PMC8005924

[28] NHLBI. National Institute of Health National Heart, Lung and Blood Institute Quality Assessment Tool for Observational Cohort and Cross-Sectional Studies. 2021. Accessed February 18, 2025. https://www.nhlbi.nih.gov/health-topics/study-quality-assessment-tools

[29] Harbord RM , Egger M, Sterne JAC. A modified test for small-study effects in meta-analyses of controlled trials with binary endpoints. Stat Med. 2006;25:3443-3457. 10.1002/sim.2380 16345038

[30] Begg CB , Mazumdar M. Operating characteristics of a rank correlation test for publication bias. Biometrics. 1994;50:1088-1101. 10.2307/2533446 7786990

[31] Halliday D , Emmanouil B, Pretorius P, et al Genetic severity score predicts clinical phenotype in NF2. J Med Genet. 2017;54:657-664. 10.1136/jmedgenet-2017-104519 28848060 PMC5740551

[32] Aboukais R , Zairi F, Baroncini M, et al Intracranial meningiomas and neurofibromatosis type 2. Acta Neurochir (Wien)). 2013;155:997-1001; discussion 1001. 10.1007/s00701-013-1692-2 23558725

[33] Li P , Wu T, Wang Y, et al Clinical features of newly developed NF2 intracranial meningiomas through comparative analysis of pediatric and adult patients. Clin Neurol Neurosurg. 2020;194:105799. 10.1016/j.clineuro.2020.105799 32229353

[34] Nowak A , Dziedzic T, Czernicki T, et al Clinical course and management of intracranial meningiomas in neurofibromatosis type 2 patients. Neurol Neurochir Pol. 2015;49:367-372. 10.1016/j.pjnns.2015.08.007 26652870

[35] Ruggieri M , Iannetti P, Polizzi A, et al Earliest clinical manifestations and natural history of neurofibromatosis type 2 (NF2) in childhood: a study of 24 patients. Neuropediatrics. 2005;36:21-34. 10.1055/s-2005-837581 15776319

[36] Champeaux-Depond C , Weller J, Resche-Rigon M. Neurofibromatosis type 2: a nationwide population-based study focused on survival after meningioma surgery. Clin Neurol Neurosurg. 2020;198:106236. 10.1016/j.clineuro.2020.106236 33002675

[37] Mohammed N , Hung YC, Xu Z, et al Neurofibromatosis type 2-associated meningiomas: an international multicenter study of outcomes after gamma knife stereotactic radiosurgery. J Neurosurg. 2022;136:109-114. 10.3171/2020.12.JNS202814 34144518

[38] Wei Z , Taori S, Mehta M, et al Primary and salvage radiosurgery for neurofibromatosis type 2–associated meningiomas. J Neurosurg. 2025;142:1125-1133. 10.3171/2024.7.JNS231815Published online November 1 39576970

[39] Thomann P , Häni L, Vulcu S, et al Natural history of meningiomas: a serial volumetric analysis of 240 tumors. J Neurosurg. 2022;137:1639-1649. 10.3171/2022.3.JNS212626 35535829

[40] Olivero WC , Lister JR, Elwood PW. The natural history and growth rate of asymptomatic meningiomas: a review of 60 patients. J Neurosurg. 1995;83:222-224. 10.3171/jns.1995.83.2.0222 7616265

[41] Islim AI , Mohan M, Moon RDC, et al Incidental intracranial meningiomas: a systematic review and meta-analysis of prognostic factors and outcomes. J Neurooncol. 2019;142:211-221. 10.1007/s11060-019-03104-3 30656531 PMC6449307

[42] Hwang WL , Marciscano AE, Niemierko A, et al Imaging and extent of surgical resection predict risk of meningioma recurrence better than WHO histopathological grade. Neuro Oncol. 2016;18:863-872. 10.1093/neuonc/nov285 26597949 PMC4864259

[43] Mirimanoff RO , Dosoretz DE, Linggood RM, Ojemann RG, Martuza RL. Meningioma: analysis of recurrence and progression following neurosurgical resection. J Neurosurg. 1985;62:18-24. 10.3171/jns.1985.62.1.0018 3964853

[44] Lemée JM , Corniola MV, Da Broi M, et al Extent of resection in meningioma: predictive factors and clinical implications. Sci Rep. 2019;9:5944-5946. 10.1038/s41598-019-42451-z 30976047 PMC6459829

[45] Pinzi V , Biagioli E, Roberto A, et al Radiosurgery for intracranial meningiomas: a systematic review and meta-analysis. Crit Rev Oncol Hematol. 2017;113:122-134. 10.1016/j.critrevonc.2017.03.005 28427502

[46] Pannullo SC , Fraser JF, Moliterno J, et al Stereotactic radiosurgery: a meta-analysis of current therapeutic applications in neuro-oncologic disease. J Neurooncol. 2011;103:1-17. 10.1007/s11060-010-0360-0 21152953

[47] Fatima N , Meola A, Pollom EL, et al Stereotactic radiosurgery versus stereotactic radiotherapy in the management of intracranial meningiomas: a systematic review and meta-analysis. Neurosurg Focus. 2019;46:E2-11. 10.3171/2019.3.FOCUS1970 31153149

[48] Habibi MA , Mirjani MS, Ahmadvand MH, et al Gamma knife stereotactic radiosurgery for neurofibromatosis 2 (NF2)-associated meningiomas; a systematic review and meta-analysis. Acta Neurochir (Wien)). 2025;167:35. 10.1007/s00701-025-06436-4 39907804 PMC11799078

[49] Pollock BE , Link MJ, Stafford SL, et al The risk of radiation-induced tumors or malignant transformation after single-fraction intracranial radiosurgery: results based on a 25-year experience. Int J Radiat Oncol Biol Phys. 2017;97:919-923. 10.1016/j.ijrobp.2017.01.004 28333013

[50] Patel TR , Chiang VLS. Secondary neoplasms after stereotactic radiosurgery. World Neurosurg. 2014;81:594-599. 10.1016/j.wneu.2013.10.043 24148883

[51] Starke RM , Yen CP, Chen CJ, et al An updated assessment of the risk of radiation-induced neoplasia after radiosurgery of arteriovenous malformations. World Neurosurg. 2014;82:395-401. 10.1016/j.wneu.2013.02.008 23403354

[52] Butts AM , Weigand S, Brown PD, et al Neurocognition in individuals with incidentally-identified meningioma. J Neurooncol. 2017;134:125-132. 10.1007/s11060-017-2495-8 28547588 PMC5544551

[53] Van Nieuwenhuizen D , Ambachtsheer N, Heimans JJ, et al Neurocognitive functioning and health-related quality of life in patients with radiologically suspected meningiomas. J Neurooncol. 2013;113:433-440. 10.1007/s11060-013-1132-4 23640137

