# Supplementary material for: A systematic review and meta-analysis of outcomes following active surveillance, surgery and radiotherapy of meningiomas in NF2-related schwannomatosis
Source: Neurooncol Adv. 2026 Feb 16;8(1):vdag022. doi: 10.1093/noajnl/vdag022 (PMC12994695; doi:10.1093/noajnl/vdag022)
Supplement: vdag022_Supplementary_Data [file vdag022_supplementary_data.zip › Supplementary Figure Legends.docx]

# Supplementary Figure Legends

## Supplementary Figure 1

## Forest plot of the weighted proportion of female patients in the cohort. The diamond represents the pooled estimate derived from fixed-effects meta-analysis.

## Supplementary Figure 2

## Forest plot of the weighted proportion of patients with multiple meningiomas. The diamond represents the pooled estimate derived from random effects meta-analysis.

## Supplementary Figure 3

## Forest plot of the weighted proportion of meningiomas that were located at the skull base. The diamond represents the pooled estimate derived from random effects meta-analysis.

## Supplementary Figure 4

## Forest plot of the weighted proportion of meningiomas that progressed to require treatment. The diamond represents the pooled estimate derived from random effects meta-analysis.

## Supplementary Figure 5

## Forest plot of the weighted proportion of patients that develop *de novo* meningiomas during active monitoring. The diamond represents the pooled estimate derived from random effects meta-analysis.

## Supplementary Figure 6

## Forest plot of the weighted proportion of “high grade” meningiomas across the resected tumors. The diamond represents the pooled estimate derived from random effects meta-analysis.

## Supplementary Figure 7

## Forest plot of the weighted proportion of recurrences in resected meningiomas. The diamond represents the pooled estimate derived from fixed-effects meta-analysis.

## Supplementary Figure 8

## Forest plot of the weighted proportion of patients that experienced complications after meningioma resection. The diamond represents the pooled estimate derived from random effects meta-analysis.

## Supplementary Figure 9

## Forest plot of the weighted proportion of patient that experienced complication after SRS treatment. The diamond represents the pooled estimate derived from fixed-effects meta-analysis.

## Supplementary Figure 10

## Forest plot of the pooled proportion of recurrences in tumors treated with SRS. The diamond represents the pooled estimate derived from fixed-effects meta-analysis.

## Supplementary Figure 11

## Forest plot of the local control rates, 3 years after SRS treatment. The diamond represents the pooled estimate derived from fixed-effects meta-analysis.

## Supplementary Figure 12

Forest plot of the local control rates, 5 years after SRS treatment The diamond represents the pooled estimate derived from fixed-effects meta-analysis.
