# Supplementary material for: A systematic review and meta-analysis of outcomes following active surveillance, surgery and radiotherapy of meningiomas in NF2-related schwannomatosis
Source: Neurooncol Adv. 2026 Feb 16;8(1):vdag022. doi: 10.1093/noajnl/vdag022 (PMC12994695; doi:10.1093/noajnl/vdag022)
Supplement: vdag022_Supplementary_Data [file vdag022_supplementary_data.zip › Supplementary Table 1.docx]

| **First Author, Year** | **Q1** | **Q2** | **Q3** | **Q4** | **Q5** | **Q6** | **Q7** | **Q8** | **Q9** | **Q10** | **Q11** | **Q12** | **Q13** | **Q14** | **Overall Score** |
| --- | --- | --- | --- | --- | --- | --- | --- | --- | --- | --- | --- | --- | --- | --- | --- |
| Aboukais R, 2013 (31) | **+** | **+** | **+** | **+** | **-** | **+** | **+** | **+** | **+** | **+** | **+** | **+** | **+** | **+** | Good |
| Birckhead B, 2016 (23) | **+** | **+** | **+** | **+** | **-** | **+** | **+** | **+** | **+** | **+** | **+** | **+** | **+** | **+** | Good |
| Champeaux-Depond C, 2020 (35) | **+** | **+** | **+** | **+** | **-** | **+** | **+** | **+** | **+** | **+** | **-** | **+** | **+** | **+** | Good |
| Dirks M, 2012 (3) | **+** | **+** | **+** | **+** | **-** | **+** | **+** | **-** | **+** | **+** | **-** | **+** | **+** | **+** | Fair |
| Evers S, 2015 (10) | **+** | **+** | **+** | **+** | **-** | **+** | **+** | **+** | **+** | **-** | **-** | **+** | **+** | **+** | Fair |
| Gao F, 2019 (24) | **+** | **+** | **+** | **+** | **-** | **+** | **+** | **+** | **+** | **+** | **-** | **+** | **+** | **+** | Good |
| Goutagny S, 2012 (5) | **+** | **+** | **+** | **+** | **-** | **+** | **+** | **+** | **+** | **+** | **-** | **+** | **+** | **+** | Good |
| Jaoude S, 2021 (7) | **+** | **+** | **+** | **+** | **-** | **+** | **+** | **+** | **+** | **+** | **+** | **+** | **+** | **+** | Good |
| Li P, 2020 (32) | **+** | **+** | **+** | **+** | **-** | **+** | **+** | **+** | **+** | **+** | **-** | **+** | **+** | **+** | Good |
| Liu A, 2015 (22) | **+** | **+** | **+** | **+** | **-** | **+** | **+** | **+** | **+** | **+** | **-** | **+** | **+** | **+** | Good |
| Mohammed N, 2022 (36) | **+** | **+** | **+** | **+** | **-** | **+** | **+** | **+** | **+** | **+** | **-** | **+** | **+** | **+** | Good |
| Nowak A, 2015 (33) | **+** | **+** | **+** | **+** | **-** | **+** | **+** | **+** | **+** | **+** | **-** | **+** | **+** | **+** | Good |
| Oyem P, 2022 (8) | **+** | **+** | **+** | **+** | **+** | **+** | **+** | **+** | **+** | **+** | **-** | **+** | **+** | **+** | Good |
| Ruggieri M, 2005 (34) | **+** | **+** | **+** | **+** | **-** | **+** | **+** | **+** | **+** | **+** | **+** | **+** | **+** | **+** | Good |
| Wei Z, 2024 | **+** | **+** | **+** | **+** | **-** | **+** | **+** | **-** | **+** | **+** | **+** | **-** | **+** | **+** | Fair |

*Supplementary Table 1: Table showing the results of the Quality Assessment performed on all included studies. (+ sign = yes, - sign = no)*
