# Supplementary figures and images for: A systematic review and meta-analysis of outcomes following active surveillance, surgery and radiotherapy of meningiomas in NF2-related schwannomatosis
Source: Neurooncol Adv. 2026 Feb 16;8(1):vdag022. doi: 10.1093/noajnl/vdag022 (PMC12994695; doi:10.1093/noajnl/vdag022)

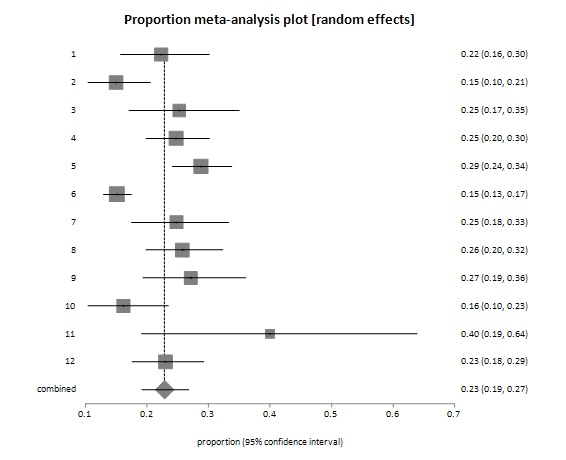

Supplement: vdag022_Supplementary_Data [file vdag022_supplementary_data.zip › Supplementary Figure 3.tiff]

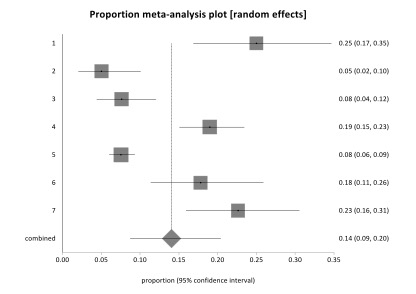

Supplement: vdag022_Supplementary_Data [file vdag022_supplementary_data.zip › Supplementary Figure 4.tiff]

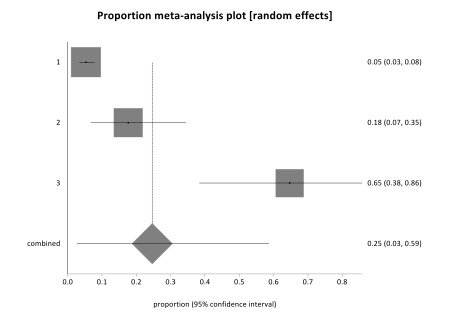

Supplement: vdag022_Supplementary_Data [file vdag022_supplementary_data.zip › Supplementary Figure 5.tiff]

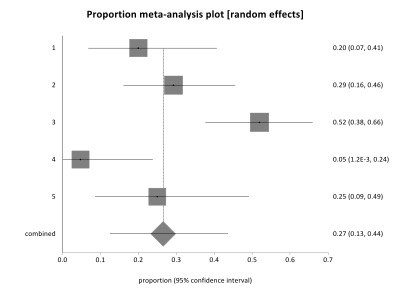

Supplement: vdag022_Supplementary_Data [file vdag022_supplementary_data.zip › Supplementary Figure 6.tiff]

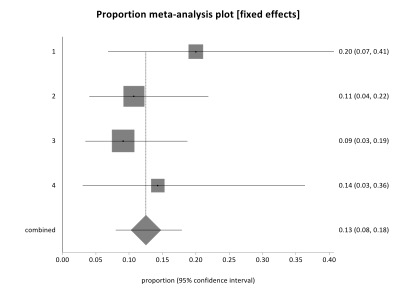

Supplement: vdag022_Supplementary_Data [file vdag022_supplementary_data.zip › Supplementary Figure 7.tiff]

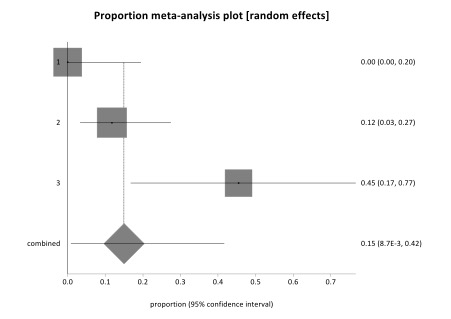

Supplement: vdag022_Supplementary_Data [file vdag022_supplementary_data.zip › Supplementary Figure 8.tiff]

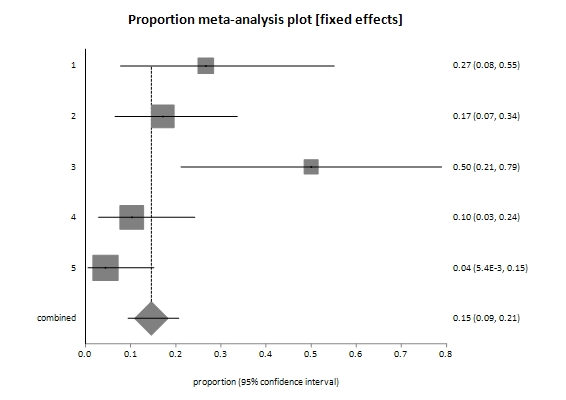

Supplement: vdag022_Supplementary_Data [file vdag022_supplementary_data.zip › Supplementary Figure 9.tiff]

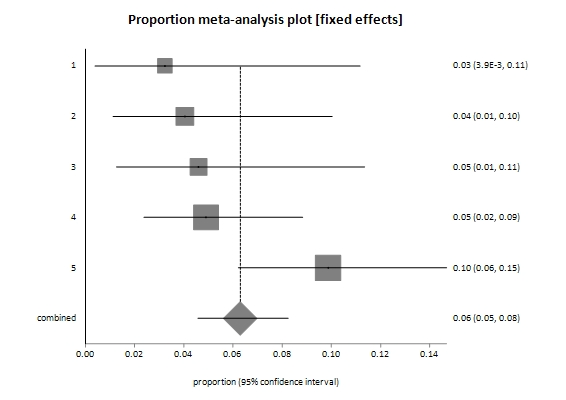

Supplement: vdag022_Supplementary_Data [file vdag022_supplementary_data.zip › Supplementary Figure 10.tiff]

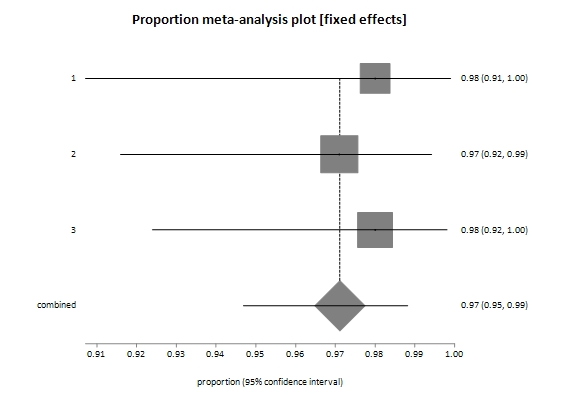

Supplement: vdag022_Supplementary_Data [file vdag022_supplementary_data.zip › Supplementary Figure 11.tiff]

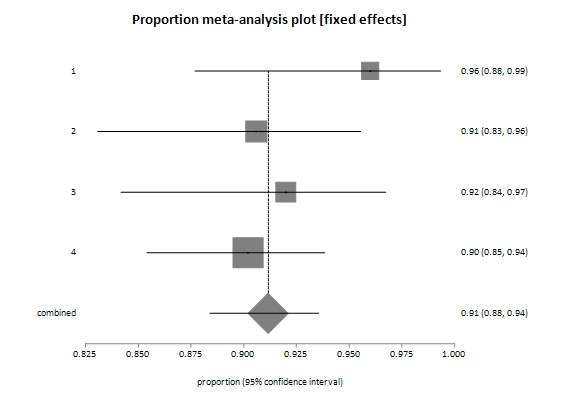

Supplement: vdag022_Supplementary_Data [file vdag022_supplementary_data.zip › Supplementary FIgure 12.tiff]

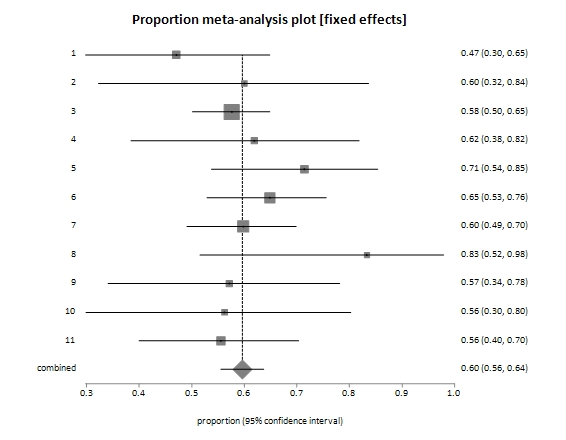

Supplement: vdag022_Supplementary_Data [file vdag022_supplementary_data.zip › Supplementary Figure 1.tiff]

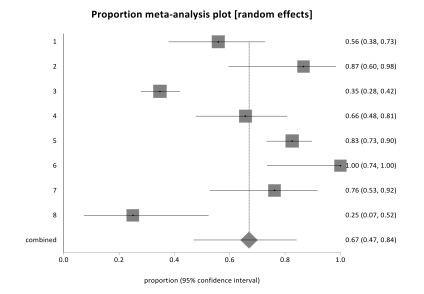

Supplement: vdag022_Supplementary_Data [file vdag022_supplementary_data.zip › Supplementary Figure 2.tiff]
